# Phylogenetic analysis of two new complete genomes of the hepatitis E virus (HEV) genotype 3 from Thailand

**DOI:** 10.1007/s11033-020-05908-3

**Published:** 2020-10-14

**Authors:** Tipsuda Chanmanee, Pravech Ajawatanawong, Suda Louisirirotchanakul, Watcharasak Chotiyaputta, Siwaporn Chainuvati, Patimaporn Wongprompitak

**Affiliations:** 1 Department of Microbiology, Faculty of Medicine Siriraj Hospital, Mahidol University, Bangkok, Thailand; 2 Division of Bioinformatics and Data Management for Research, Faculty of Medicine Siriraj Hospital, Mahidol University, Bangkok, Thailand; 3 Division of Gastroenterology, Department of Medicine, Faculty of Medicine Siriraj Hospital, Mahidol University, Bangkok, Thailand; 4 Department of Immunology, Faculty of Medicine Siriraj Hospital, Mahidol University, Bangkok, Thailand

**Keywords:** Hepatitis E virus, HEV genotype, HEV genotype 3, Complete genome sequence, Phylogeny

## Abstract

Hepatitis E virus (HEV) is a causative agent of acute viral hepatitis globally. Evolutionary phylogeny classifies the HEV into eight genotypes that correlate with the viral transmission. Only four genotypes have been proven to be responsible for transmission in humans. However, there has been no report on the genomics and genotyping of HEV in Thailand during the past ten years. Here, we identified the genotype distributions of the Thai isolates of HEV and we sequenced two HEV genomes. We screened for 18 Thai isolates of HEV from Siriraj Hospital in Bangkok, from 2014–2016. The HEV genomes were sequenced from the serum and feces of a patient. The results showed that all Thai isolates of HEV were identified as genotype 3 (HEV-3). The ORF2 and genome phylogenies suggested two subgenotypes, called 3.1 and 3.2. The Thai isolates of HEV were frequently found in the subgenotype 3.1. The genome sequences of the two Thai isolates of HEV from the serum and fecal samples of the same patient showed 91% nucleotide similarity with the HEV genotype 3. Comparisons between the HEV genome and the ORF2 phylogenies illustrated that the ORF2 tree can be used to identify HEV genotypes, but it has less phylogenetic power for the HEV evolution. The two new genome sequences of HEV-3 from Thailand could contribute valuable information to the HEV genome study. (226 words)

## Introduction

Hepatitis E virus (HEV) or *Orthohepevirus* A is an emerging viral infection that causes acute hepatitis, which is considered as leading to a low rate of mortality and not developing into chronic hepatitis [1]. However, the incidence of chronic hepatitis appears to be increasing among immunocompromised patients who have had an organ transplant, in developing countries, particularly industrial countries [2, 3]. The virus transmits when people consume contaminated food or water [1]. There are two main laboratory-detection methods for HEV. One is the molecular-based method using reverse transcription-PCR, which is generally targeted at the viral capsid protein. Another is the immunological-based method for the detection of IgM and IgG against HEV antigens [4].

HEV is classified as a member in the genus *Orthohepevirus* of the family *Hepeviridae*. The structure of HEV particle is comprised of a positive, single-stranded RNA genome packed inside the icosahedral capsid proteins [5]. Genome of the virus has ~ 7.2 bases in length, including a 5′-untranslated region (5′-UTR), three open reading frames (ORFs), and a 3′-untranslated region (3′-UTR). The ORF1 contains at least four nonstructural proteins, including a methyltransferase, a cysteine protease, a helicase, and an RNA-dependent RNA polymerase [6]. ORF2 is laid behind the ORF1 and encodes a capsid antigen protein. The ORF3, the smallest ORF, which is overlapped with the ORF2, encoded a protein with an unknown function [6].

Phylogenetic analysis of the HEV genomic sequences illustrated the virus are highly diverged into eight genotypes. The HEV genotype 1 and 2 are closely related genotypes [7, 8]. The genotype 1 can be found in Asia, Africa, and South America, whereas the genotype 2 are found in Mexico, Nigeria, and Chad [9]. The viruses in both genotypes mainly infected humans, so-called “anthropotropic genotypes”, and transmitted by contamination in supply water [8]. The HEV genotype 3 are spread globally. The HEV genotype 4 is found to be limited in East Asia, including Japan, China, and South Korea [10, 11]. The HEV in both genotype 3 and 4 are found as sporadic infection in human, pigs, and other animals [6, 10]. Chronic hepatitis may develop in immunosuppressed patients infected with HEV genotypes 3 or 4 [6]. The viral transmission occurs by ingestion of raw or undercooked meat from infected animals, so-called “enzootic genotypes”. Unlike the major genotypes, the HEV genotype 5–8 are small groups and found in animals. HEV genotypes 5 and 6 are the isolates from wild boars in Japan; HEV genotype 7 was identified from dromedary camels in the United Arab Emirates and other countries in the Middle East; HEV genotype 8 comes from camels and circulates in many regions in China [6, 10–13].

For this study, we observed HEV RNA among 18 acute HEV patients in Thailand from 2014 to 2016. In fact, there are few studies of HEV phylogenies in Thailand particularly in a comparison between ORF2 and genome phylogenetic trees. This observation was done in Thailand after the last survey, which was done about a decade ago [14, 15]. The partial ORF2 were sequenced and we performed phylogenetic reconstruction for HEV genotype identification. Of those 18 patients, only one had adequate serum and fecal specimens for whole-genome sequencing. The complete HEV genome sequences were subjected to phylogenetic reconstruction to determine of the HEV genome evolution, as well.

## Materials and methods

### Subjects and samples

Hepatitis patients who admitted to the hospital at the Faculty of Medicine Siriraj Hospital, Mahidol University in Bangkok from 2014 to 2016, were investigated for HEV. All patients tested positive for hepatitis, specifically due to their elevation of two liver enzymes, aspartate aminotransferase (AST) and alanine transaminase (ALT). The serum and feces were collected from all 33 hepatitis patients to investigate the antibodies against HEV and viral HEV RNA. The serological markers with anti-HEV IgM and IgG were detected by indirect ELISA (DIA.PRO, Milan, Italy) based on the HEV-specific antigens of four HEV genotypes. Sera and fecal specimens were used to detect HEV RNA by PCR of a 0.9-kb-region in the ORF2. However, the serum and fecal samples from all these patients were taken on the same day.

Only one sample (from patient number TH-hu-45) had enough RNA for a genome sequencing. This person was 68 years old, and had developed an abnormal liver function, with an increase in three liver markers, including alanine transaminase (ALT) at the level of 640 U/L; aspartate transaminase (AST) at the level of 165 U/L; and alkaline phosphatase (ALP) at the level of 310 U/L. The Ethics Committee approved the protocol used in this study for Research in Humans of the Faculty of Medicine Siriraj Hospital, Mahidol University (IRB no. Si 699/2014). Written informed consent was obtained from all the participating patients.

### Amplification of HEV RNA for detection and nucleotide sequencing

The HEV RNA was extracted from 140 µl of the specimen using a QIAmp Viral RNA Mini Kit (QIAGEN, Hilden, Germany). The complementary DNA (cDNA) was synthesized from HEV RNA by reverse transcription with a Superscript III First-Strand Synthesis System (Invitrogen, Carlsbad, CA, USA) using a random hexamer. The cDNA was used for the determination and sequencing of HEV ORF2. For HEV determination, the first round nested PCR of the ORF2 fragment was amplified by HE361 (5′-GCRGTGGTTTCTGGGGTGAC-3′) and HE364 (5′-CTGGGMYTGGTCDCGCCAAG-3′) primers. The second-round PCR then followed the PCR amplicons with HE366 (5′-GYTGATTCTCAGCCCTTCGC-3′) and HE363 (5′-GMYTGGTCDCGCCAAGHGGA-3′) primers [15, 16]. The PCR contained 5 µL of DNA template, 5 µL of 10xPCR buffer, 4 µL of 25 mM dNTP, 0.5 µL of 5 U/µL EX TaKaRa *Taq* (TaKaRa Bio, Inc., Shiga, Japan), 20 pmol of forward and reverse primers, and was adjusted a final volume of 50 µL by DNase-RNase-free distilled water. PCR was performed for 35 cycles of 94 °C for 30 seconds; 55 °C for 30 seconds; and 72 °C for one minute. The second round was amplified for 25 cycles under the same amplification conditions. The expected size of the final PCR amplicon was 137 base pairs (bp) in length.

In case of nucleotide sequencing of HEV ORF2, we needed a longer PCR amplicon (919 bp), which contained enough phylogenetic signals in the sequences. The entire cDNA samples were performed semi-nested PCR using HE6209F (5′-CAGCCACACGTTTYATGAAGGA-3′) and HE3′ (5′-TTTTTTTTCCAGGGAGCG-3′) as the outer primers. The inner primers were HE6326F (5′-CGACAGAATTGATTTCGTCGGC-3′) and HE3′ primers. The PCR products of the first and second round had a size of 1036 bp and 919 bp, respectively. The PCR amplicons were purified by QIAquick Gel Extraction Kit (QIAGEN).

### HEV genome sequencing and assembly

To sequence the HEV genome, 12 sets of oligonucleotide primers (Table 1) were designed overlappingly along the genome as the genome sequencing strategy. After we determine the concentration of HEV cDNA left-over from RNA determination, one left over cDNA from a patient, TH-hu-45, had enough concentration for genome sequencing. The cDNA from this fecal sample was coded as TH-hu-F45-1. Fortunately, we also had cDNA from the serum sample (TH-hu-S45-1) of the same patient. The fecal and serum samples were taken from the subject on the same day.

**Table 1:** A list of the 12 oligonucleotide primer sets used for hepatitis E virus (HEV) whole-genome-sequence amplification

Set	Outer/inner	Primer	Oligonucleotide primer sequence	Region	Position	Product size (bp)	Ta (°C)
1	Outer	HE5’	GTC GAT GCC ATG GAG GCC	5′UTR/ORF1	1–18	543	54
		HE52	CCG AGG GCC AAA GGT CAT G		525–543		53
	Inner	HE5’	GTC GAT GCC ATG GAG GCC		1–18	473	54
		HE452	CCC ATC GAA GCA GTA TGT GCG		452–473		53
2	Outer	HE01	AAG GCT CCT GGC ATT ACT ACT	ORF1	48–70	955	53
		HE02	AAR AGC ATR AGC CGR TCT CA		984–1003		51
	Inner	HE01	AAG GCT CCT GGC ATT ACT ACT		48–70	901	53
		HE03	GTA GAG CAR GCT GAK GGR AA		930–949		51
3	Outer	HE741F	TGG ATC CGC ACC ACT AAA ATA	ORF1	741–761	1173	52
		HE1895Rst	CGG TRC AAT CCA RGC CAT TA		1895–1914		52
	Inner	HE741F	TGG ATC CGC ACC ACT AAA ATA		741–761	1000	52
		HE1722Rn	AAG RCC TTA TTC CCG AGC AC		1722–1741		56
4	Outer	HE1498Fst	TGG TTG GTG ACT GTG GCC AT	ORF1	1498–1517	877	55
		HE2293Rst	TGG TGT RGG CGG CTT ACG G		2357–2375		56
	Inner	HE1560Fn	CCC GCC CAC CTT GAT GTT TC		1560–1579	815	55
		HE2293Rn	TGG TGT RGG CGG CTT ACG G		2357–2375		56
5	Outer	HE1990F	ACA ATA GGT TCA CCC AGC GCC	ORF1	1990–2010	1085	55
		HE3054Rst	GTC GCC AAC TAT TGC GGA GCT C		3054–3075		55
	Inner	HE1990F	ACA ATA GGT TCA CCC AGC GCC		1990–2010	1016	55
		HE2985Rn	TAG ACT TTC CGG AGC CGG GAA C		2985–3006		55
6	Outer	FfrR4st	GTC GAT GCC ATG GAG GCC	ORF1	2795–2815	1007	54
		FfrR4n’	CCG AGG GCC AAA GGT CAT G		3783–3802		55
	Inner	FfrR4st	GTC GAT GCC ATG GAG GCC		2795–2815	944	54
		RfrR4	CCC ATC GAA GCA GTA TGT GCG		3783–3802		55
7	Outer	HE3560Fst	TGC GCA TGC TAT CGT TGC ACT	ORF1	3560–3580	956	55
		HE4497R	GGC ATG CCA CAY TCC TCC AT		4497–4516		55
	Inner	HE3664Fn	ACT TCT TCC TGG CTG GTG GTG A		3664–2685	852	55
		HE4497R	GGC ATG CCA CAY TCC TCC AT		4497–4516		55
8	Outer	HE4263Fst	GCA TAT CGG CTT GGA GTA AGA C	ORF1	4263–4285	816	52
		HE5058R	AGT GGG CCT TTC CAT CAG CAA T		5058–5079		55
	Inner	HE4332Fn	ATT CTA GCT TTG CTC CCG CCC		4332–4352	747	55
		HE5058R	AGT GGG CCT TTC CAT CAG CAA T		5058–5079		55
9	Outer	HE4974F	CGC AGG TTT GTG TTG ATG TTG T	ORF1/ORF2	4974–4996	677	54
		HE5631Rst	GGG GCG GCA TAC AAG ACA AGA		5631–5651		55
	Inner	HE4974F	CGC AGG TTT GTG TTG ATG TTG T		4974–4996	610	54
		HE5563Rn	TAT ACT GGC GGC GCA AGA TAG C		5563–5584		55
10	Outer	HE5204F	TCT TCG TGC TTC TGC CTA TGC TG	ORF2	5204–5226	972	55
		HE6159Rst	CCA CGA CGC AAA CGG TGT		6159–6176		54
	Inner	HE5204F	TCT TCG TGC TTC TGC CTA TGC TG		5204–5226	855	55
		HE6040Rn	CCG GTA TAG GGC GTG TTG GT		6040–6059		55
11	Outer	ORF2F2	CHA TYT CTA TYT CYT TYT GGC C	ORF2	5890–5912	955	56
		ORF2R2	GTA GTC TGR TCA TAY TCA GCV GC		6703–6725		55
	Inner	ORF2F2	CHA TYT CTA TYT CYT TYT GGC C		5890–5912	901	56
		Cona1M	TCT TGT TCR TGY TGG TTR TCR TAR TC		6524–6499		55
12	Outer	HE6209Fst	CAG CCA CAC GTT TYA TGA AGG A	ORF2/3′UTR	6209–6230	1036	56
		HE3prime	TTT TTT TTT TTT TTT TTT TCC AGG GAG CG		7220–7245		55
	Inner	HE6326Fn	CGA CAG AAT TGA TTT CGT CGG C		6326–6347	919	56
		HE3prime	TTT TTT TTT TTT TTT TTT TCC AGG GAG CG		7220–7245		55

The PCR amplification conditions were varied by the melting temperature of each pair of primers. The PCR amplicons for each pair of primers were purified using the QIAquick Gel Extraction Kit (QIAGEN). The purified PCR amplicons were cloned into the pCRTM2.1-TOPO vector (Invitrogen). The nucleotide sequencing was performed using Sanger’s sequencing by M13 forward and reverse primers (Macrogen, Seoul, South Korea). All PCR amplicons were sequenced in both strands to prevent sequencing errors. All sequence electropherograms were read and edited manually. The edited sequences from both strands were confirmed by BLASTN and consensus sequences by the SeaView program version 5.0.4 [17]. The 12 HEV fragments were assembled manually.

### Phylogenetic analyses

The 19 sequences of the HEV partial ORF2 sequences (accession number: MH427090–MH427106, KY232312, KY232313) and 43 sequences (accession number: AB369687, EU723513, EU360977, KJ873911, AB290313, AB248521, AF455784, FJ705359, FJ998008, AB290312, JQ953664, JQ013794, AF082843, AP003430, AY115488, AB369689, AB220974, GU119961, AB108537, AB369688, AB074915, DQ450072, AB197673, AJ272108, AY723745, DQ279091, AB602441, AB856243, AB573435, M73218, JF443721, X98292, AY230202, AY204877, D11092, M74506, KJ496143, KJ496144, JQ013791, FJ906895, KJ013415, LC177788, and NC038504) of HEV ORF2 in the other study [18] were used as the dataset for a phylogenetic analysis of the partial ORF2 sequences. All sequences were aligned by the MUSCLE program [19, 20] via the SeaView program [17]. The multiple sequence alignment (MSA) was trimmed only at 5′- and 3′-terminals. The size of the final matrix was 568 characters × 62 taxa. The phylogenetic analyses were performed by the maximum likelihood (ML) method using the PhyML program [21] via the SeaView program [17], and Bayesian Inference (BI) method using MrBayes version 3.2.7 [22]. The ML tree was run with the GTR + I + G model. The bootstrap values were calculated from 1000 replications of pseudodata. The BI tree was run with 1,000,000 generations or until the average standard deviation of the split frequencies fell below 0.01. The trees were sampled with every 100 generations, and a total of 20,000 trees were generated with the initial sample. The trees were edited with FigTree version 1.4.4 [23].

The dataset of the HEV genome sequences for phylogenetic reconstruction contains two complete sequences from our study (accession number: KY232312 and KY232313) and another 95 genome sequences from the other studies (accession numbers: AB074918, AB074920, AB089824, AF082843, AY115488, AP003430, AB189070, AB091394, AB073912, AB369689, AB740232, KC618403, KC618402, FJ705359, KJ701409, FJ998008, JQ013794, AB290312, JQ953664, AB369687, FJ653660, AB291961, EU375463, FJ956757, KC166971, EU723513, EU360977, AB290313, AB248521, KJ013415, AB248522, HM055578, FJ998015, KF922359, JQ013795, AF455781, FJ906895, JQ013791, KJ496143, KJ496144, AF076239, AF459438, X99441, AF051830, M73218, D10330, AF185822, JF443721, L08816, M94177, L25595, AF444003, D11092, D11093, X98292, AY230202, AY204877, M74506, AB161791, AB220972, AB220973, AB220975, AB074917, AB161717, AB220977, AB220979, AB220976, AB099347, AB193176, AB193178, AB193177, AB200239, AB220971, AB097811, AB080575, AB074915, GU119961, AB220974, AB108537, AB369688, DQ279091, EU676172, AB253420, AJ272180, AY594199, AB197674, EF077630, AB197673, AY723745, DQ450072, AB602441, AB856243, AB573435, LC177788, NC038504) [15, 24–73]. The dataset was aligned with the same method for analysis of the ORF2 region. Phylogenetic analysis was done with both ML and BI, using the same setting parameters as the ORF2 phylogeny.

## Results

### Serological and molecular detection of HEV in Thai patients

The 33 serum samples were collected from 33 patients with hepatitis, who showed an abnormal liver function, based on the enzymes, aspartate aminotransferase (AST) and alanine transaminase (ALT). All serum samples were screened for IgM and IgG by the ELISA method. Of the 33 patients, only 20 samples (60.6%) were diagnosed as HEV infection (Table 2) by either positive for IgM or IgG, or positive for both. The sample TH-hu-FPP was negative for IgM but positive for IgG.

**Table 2:** Samples and sequences used in this study: each sample ID referrs to individual hepatitis patients

Sample ID	Anti-HEV		HEV RNA		Accession number
	IgM	IgG	In serum	In feces	
TH-hu-FJN	+	ND	−	+	MH427099
TH-hu-FNP	+	+	+	+	MH427105
TH-hu-FYS	+	+	−	+	MH427098
TH-hu-FUD	+	+	−	+	MH427100
TH-hu-FKP	+	+	+	+	MH427106
TH-hu-FSN	+	+	+	+	MH427095
TH-hu-FOG	+	+	+	+	MH427103
TH-hu-FTM	+	ND	+	+	MH427096
TH-hu-FLR	+	+	−	+	MH427104
TH-hu-F40	+	+	+	+	MH427091
TH-hu-FPP	-	+	+	+	MH427092
TH-hu-FKS	+	+	+	+	MH427090
TH-hu-FSP	+	ND	+	+	MH427097
TH-hu-F45-1	+	ND	+	+	KY232312, KY232313
TH-hu-FTK	+	+	−	+	MH427094
TH-hu-FTS	+	+	+	+	MH427102
TH-hu-FVS	+	+	+	+	MH427093
TH-hu-F47	+	+	+	+	MH427101

The anti-HEV, both IgM and IgG, were detected in the serum specimens. The HEV RNA was detected from serum and fecal samples. HEV in the serum and fecal specimens from patient TH-hu-F45-1 was chosen to perform genome sequencing. The accession number KY232312 and KY232313 refer to the HEV from serum and feces, respectively

*ND* not done

The 20 HEV patients were then tested for HEV RNA, using their serum and feces. Only 18 patients were positive for the reverse-transcription PCR of the HEV capsid gene in feces. Of those 18 fecal HEV-positive patients, only 13 (72.2% of 18 people) had the HEV RNA detectable in their serum (Table 2). This result suggests that the detection of HEV RNA in feces has more potential than it does through serum.

### Phylogenetic-based HEV genotype identification

**Fig. 1:**
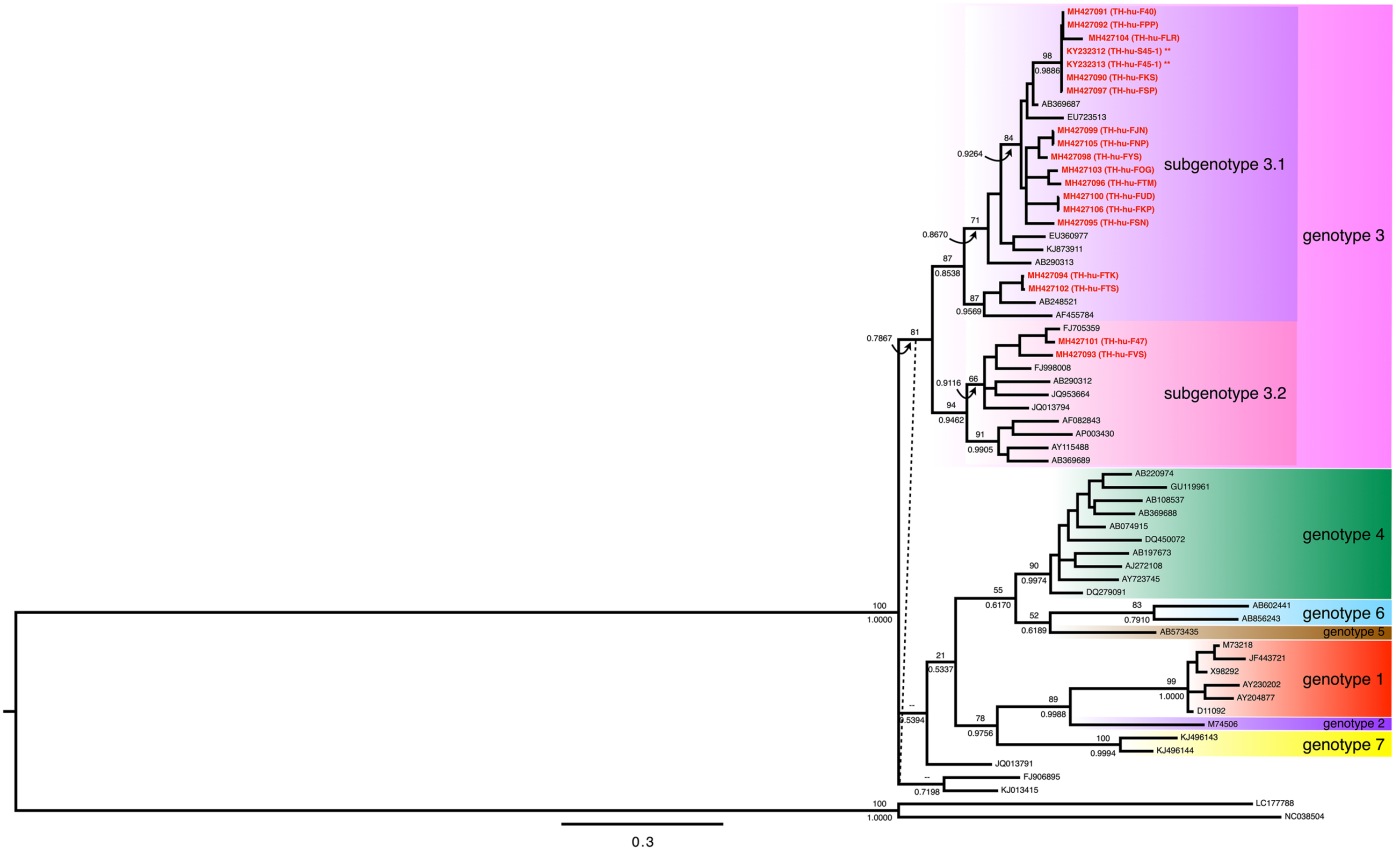
Bayesian inference (BI) phylogeny of 62 partial nucleotide sequences in the ORF2 (a capsid gene containing ORF) of HEV. The topology of the BI phylogeny was slightly different from the phylogeny reconstructed by the maximum likelihood method. The dashed lines show the differences in positions between those phylogenies. The statistical support is shown at the deep branches that connect to the specific nodes. The number above the branch represent the bootstrap value calculated from 1000 replications of the maximum-likelihood method, while the number underneath the same branch represents the posterior probability calculated by the Bayesian inference-phylogenetic method. The operational taxonomic units (OTUs) with a red color show the accession numbers of the HEV sequences in this study, and the isolate identification numbers are shown in the brackets. The isolates that were selected to perform the genome sequences are marked with asterisks (**). The black OTUs show the accession number of HEV sequences from the other study [18]. The HEV genotypes and subgenotypes are labeled in different colors. Scale bars indicate the distance in nucleotide substitution per site

We performed the Sanger’s sequencing of the partial capsid gene, which was located on the open reading frame 2 (ORF2) of the HEV genome, from the 19 reverse transcription PCR positive amplicons, but from 18 HEV patients. That was because the patient “TH-hu-45” had reverse transcription PCR positive in both serum and fecal samples. So, the 19 capsid sequences from the Thai HEV and the other 43 HEV from the other study [18] were then aligned and trimmed the incomplete sequence regions at both ends of the multiple sequence alignment (MSA). The alignment matrix of 566 characters × 63 taxa was subjected to the molecular phylogenetic analyses using the maximum likelihood (ML) and Bayesian Inference (BI) methods. The tree topologies between the ML and the BI phylogenies were almost identical. The conflict topology was presented in the clade of FJ906895 and KJ013415, which appeared to be the sister clade of the genotype 3 in the ML tree. The Bayesian phylogeny of these sequences illustrated at least five monophyletic clades correlated with the HEV genotypes (Fig. 1). The HEV genotypes 3 was appeared to be the sister clade of the remaining genotype (e.g., genotype 1, 2, 4, 5, 6, and 7). The HEV non-genotype 3 were clustered in a monophyletic group with two subclades. The former showed a cluster of the genotypes 4, 5, and 6. The single taxon belonging to genotype 5 was the sister taxon of genotype 6, and genotype 5 and 6 were the sister clade of genotype 4 (Fig. 1). The latter illustrated the cluster of HEV genotypes 1, 2, and 7. The single taxon of genotype 2 was a sister taxon of genotype 1, and they were sister clades of HEV genotype 7 (Fig. 1).

All 19 Thai isolates of HEV were identified as the HEV genotype 3 (Fig. 1). The HEV genotype 3 appeared to be the largest monophyletic clade of HEV. This clade was further separated into two subclades; subclade 3.1 and subclade 3.2. Interestingly, the Thai isolates of HEV were present in both subclade 3.1 and 3.2. Seventeen sequences were identified as being in the subgenotype 3.1, whereas the other two sequences belonged to the subgenotype 3.2. There were three monophyletic groups of nucleotide sequences of the Thai isolates of HEV in the subgenotype 3.1: (i) the first clade was comprised of seven sequences, including TH-hu-F40, TH-hu-FPP, TH-hu-FLR, TH-hu-S45-1, TH-hu-F45-1, TH-hu-FKS, and TH-hu-FSP; (ii) the second clade was comprised of eight sequences, including TH-hu-FJN, TH-hu-FNP, TH-hu-FYS, TH-hu-FOG, TH-hu-FTM, TH-hu-FUD, TH-hu-FKP, and TH-hu-FSN; and (iii) the third clade was comprised of two sequences, TH-hu-FTK and TH-hu-FTS (Fig. 1). The two nucleotide sequences, TH-hu-F47 and TH-hu-FVS, in the subgenotype 3.2 formed paraphyletic relationship on the tree (Fig. 1).

### Genome sequences of the Thai isolates of HEV

**Fig. 2:**
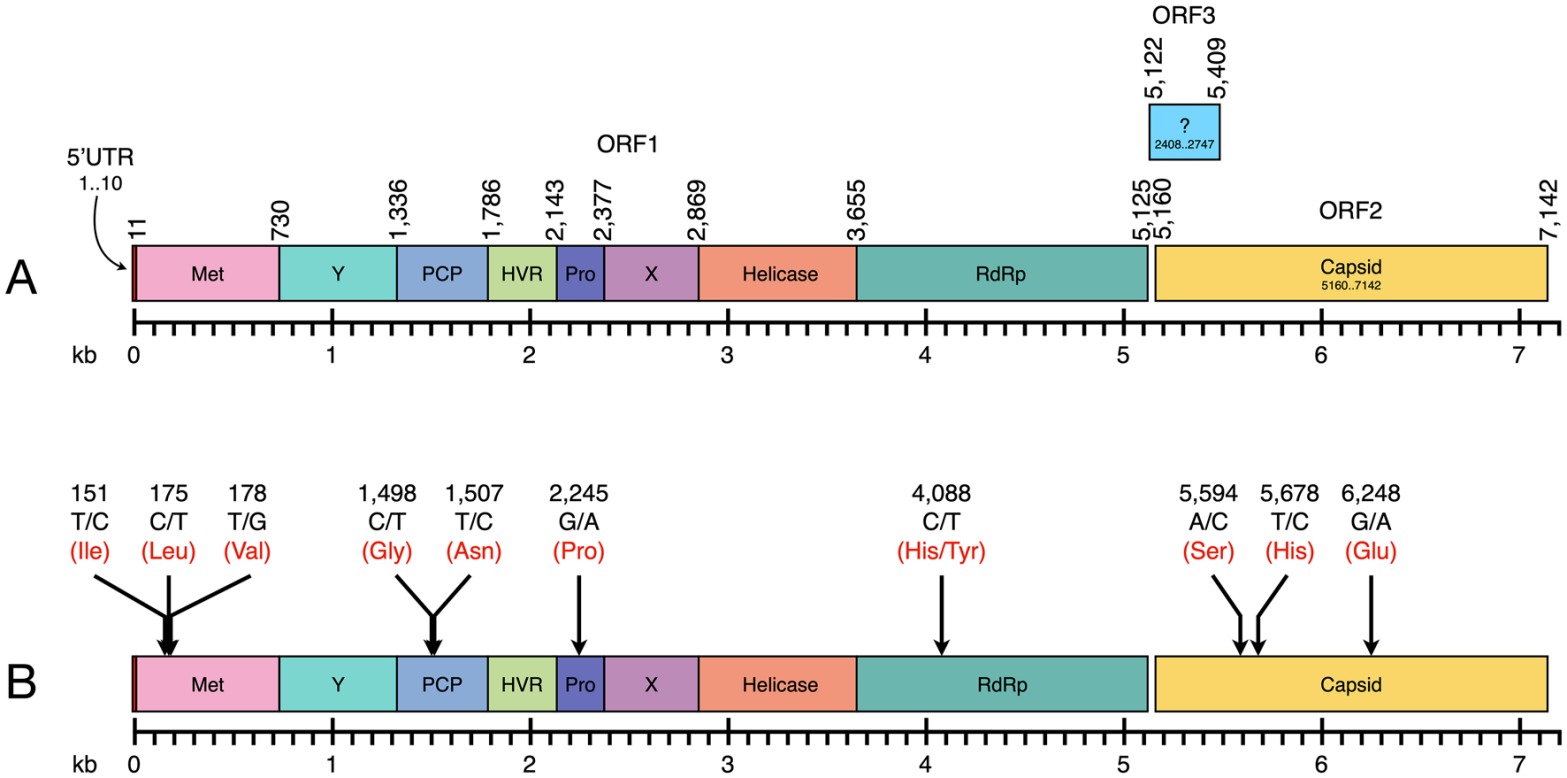
Genome organization of the Thai isolates of HEV (KY232312 and KY232313) and mutation events between those two genomes: the HEV genomes from serum and feces were identical in size and organization (**a**). The total length of the HEV genomes was 7142 bp, organized in three ORFs—1, 2, and 3. The ORF1 encoded a nonstructural polyprotein which contained at least eight regions, including a methyltransferase (Met), a Y domain (Y), a papain-like cysteine protease (PCP), a hypervariable region (HVR), a proline-rich region (Pro), a protein with an unknown function (X), a helicase (Helicase), and an RNA-dependent RNA polymerase (RdRp). The ORF2 encoded a single capsid gene (Capsid), and the ORF3 also carried a single protein with unknown function. The numbers on the genome diagram represent the boundary of each protein in the genome. Nucleotide substitution sites on the two genomes are shown by arrows and are labeled in black numbers (**b**). The labels at each arrow tail represent the nucleotide substitution site, pattern of nucleotide-substitution, and amino acid. The nucleotide in the HEV genome isolated from serum (KY232312), and feces (KY232313) are separated by a slash (/). The amino acids encoded from the mutation position are shown in red. Regarding the non-synonymous substitution, the amino acids in the different genomes are separated by a slash (/)

We attempted to sequence the HEV genome from all HEV-positive patients, but, unfortunately, only two samples from one patient (TH-hu-45) had enough RNA to perform the genome sequencing of HEV. The serum and fecal samples were then sequenced along the entire coding regions (a nearly complete genome) by direct sequencing of the 12 amplified fragments along the HEV genome. After these were manually assembled, we derived two scaffold genomes. One was the HEV isolate TH-hu-S45-1 (accession number KY232312), which derived from a serum sample, and the other was TH-hu-F45-1 (accession number KY232313), the HEV from the fecal sample. The genome size of these two isolates appeared to be the same size of 7235 nucleotides in length (Fig. 2a).

Both genomes were comprised three open reading frames (ORFs). The ORF1 had 5115 nucleotides in length (position 11–5125 in the genome sequence), which was encoded for a polyprotein that was cleaved into eight polypeptides, starting from a viral methyltransferase enzyme; a Y domain; a papain-like cysteine protease; a hypervariable region; a proline-rich region; a protein with unknown function; a helicase; and an RNA-dependent RNA polymerase (Fig. 2a). The ORF2 had 1983 nucleotides in length at position 5160–7142, which located at the 3′-terminal of the genome. This region was coded for 660 amino acid residues of the viral capsid protein. The ORF3 was the smallest ORF that overlapped with the ORF1 and ORF2 on the HEV genome (Fig. 2a). It contained 369 nucleotides at the position 5122–5490, and was coded for a protein with an unknown function.

Genome comparisons between the HEV isolated from serum and feces showed 99.8% similarity among nucleotide sequences and 99.9% identity of the amino acid sequences. The total of ten nucleotide-substitutions occurred between these two isolates of HEV. Seven of ten substitutions were detected on the ORF1, while the other three positions were detected on the ORF2 (Fig. 2b). In the ORF1, three synonymous substitutions occurred in the viral methyltransferase gene (positions 151, 175, and 178), two synonymous substitutions occurred in the protease gene (positions 1498 and 1507), and one synonymous substitution occurred in the proline-rich protein (position 2245). Only one mutation at the position 4088 was identified as a non-synonymous substitution, which was presented as histidine (H) in the HEV isolated from serum, but as tyrosine (Y) in the HEV isolated from feces of the same patient. The other three synonymous substitutions (positions 5594, 5678, and 6248) were found on the capsid protein (ORF2). There was no nucleotide substitution detected on the ORF3.

### Genome-scale phylogeny of HEV

**Fig. 3:**
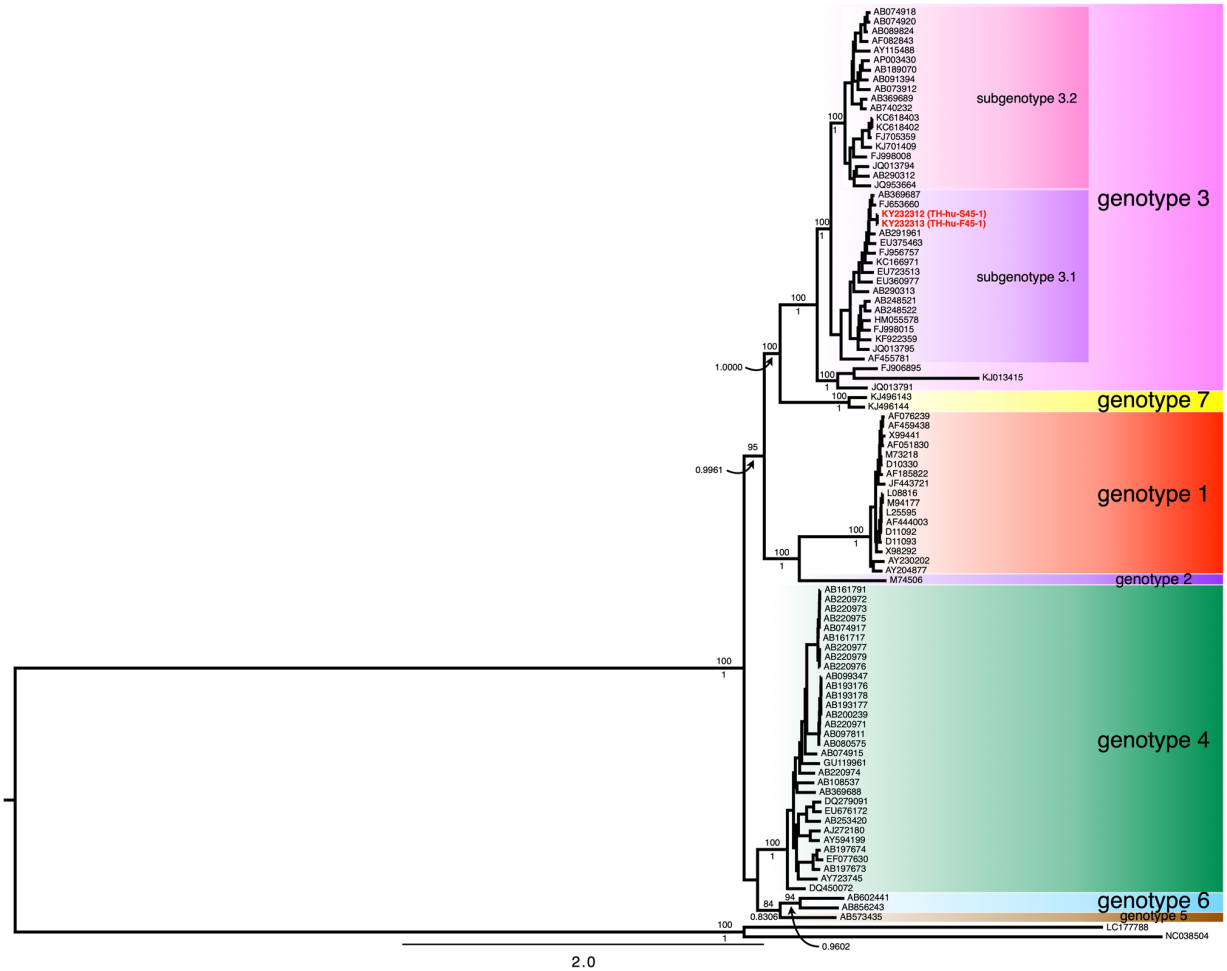
The Bayesian inference (BI) phylogeny of 97 partial genome sequences of HEV, including the two isolates from this study. The phylogenetic analyses were reconstructed by ML and BI methods. The tree topologies were identical between both the ML and BI phylogenies. Statistical supports of the branches are shown on the deep branches. The number above the branch represents the bootstrap value calculated from 1000 replications of the maximum-likelihood method, while the number underneath the same branch represents the posterior probability calculated by the Bayesian inference phylogenetic method. The OTUs in red show the accession numbers of the HEV genome sequences in this study, and the isolate identification numbers are shown in brackets, whereas the OTUs in black show the accession number of HEV sequences from the other study [15, 24–73]. The HEV genotypes and subgenotypes are labeled with different-colored boxes. Scale bars indicate the distance in nucleotide substitutions per site

Phylogenetic reconstruction of two HEV genome sequences isolated from a Thai patient and the 95 genome sequences from the other studies were performed using the ML and BI methods. Tree topologies at the deep branching of the ML and BI phylogenies were identical (Fig. 3). Obviously, the genome phylogenies clustered HEV by the genotypic patterns of the HEV. Comparisons between the genome and the ORF2 phylogenies illustrated three different points. First, the basal split separated HEV into two major clades, which contained the HEV genotypes 4, 5, and 6 in one clade and the remaining genotypes in another clade (Fig. 3). However, the basal split in the ORF2 phylogeny appeared to be the polytomy of three clades, including (i) the HEV genotype 3, (ii) the unclassified HEV genotype 3 (FJ906895 and KJ013415) isolated from rabbits, and (iii) the remaining HEV genotypes and JQ013791—an isolate of HEV genotype 3 (Fig. 1). Second, HEV genotype 3 formed a monophyletic group in the genome phylogeny (Fig. 3), but polyphyletic in the ORF2 phylogeny (Fig. 1). That is because the three HEV isolated from rabbits (FJ906895, KJ013415, and JQ013791) were excluded from the HEV genotype 3 clade. Nevertheless, in the ORF2 phylogeny, the sequences of HEV genotype 3 isolated from human formed a monophyletic group, but the branching positions of the HEV sequences isolated from rabbits were incongruence between the ML and BI phylogenies (Fig. 1). Third, the genotype 3 clade in both genome and ORF2 phylogenies was split into two subgenotypes, called 3.1 and 3.2. These revealed exhibited that the ORF2 phylogeny had low phylogenetic discriminatory power for HEV genotyping than the genome phylogeny, particularly for the HEV genotype 3. Therefore, the phylogenetic identification of HEV genotypes based on the genome-scale data can be more reliable than the power of the partial ORF2 sequences. Both Thai isolates of HEV were identified as HEV genotype 3, subgenotype 3.1 (Fig. 3).

## Discussion

We detected HEV RNA from 18 fecal specimens from a total of 20 Thai patients, who developed IgM or IgG against HEV from 2014 to 2016 (Table 2). All 18 HEV RNA-positive samples were then sequenced for partial ORF2 region (919 bp), which encoded a part of viral capsid protein. The phylogenetic analysis revealed that all Thai isolates of HEV belonged to the HEV genotype 3 (Fig. 1, 3). The HEV genotype 3 was further divided into two subgenotypes, 3.1 and 3.2. Only two isolates of the HEV in this study were identified as subgenotype 3.2, whereas the other isolates belonged to the subgenotype 3.1 (Fig. 1). Only one HEV patient had enough concentration of RNA in the fecal and serum specimens for performing whole genome sequencing. Therefore, only two HEV genomes were successfully sequenced from the serum (KY232312) and feces (KY232313) of the same patient. Both HEV genomes illustrated high similarity to the typical HEV genome (Fig. 2a). However, there were ten nucleotide substitutions found in both genomes. A single non-synonymous substitution occurred at position 4088, while the other nine synonymous substitutions were detected along the genome (Fig. 2b). Both Thai HEV genome were identified as the HEV genotype 3, and the subgenotype 3.1 (Fig. 3). The genome tree appeared to have more phylogenetically informative than the ORF2 phylogeny at least for HEV genotype identification and because of the monophyly of the genotype 3 (Figs. 1, 3).

The immunological diagnosis for confirming acute HEV infection typically relies on the presence of IgM. In our study, we found IgM in all patients’ sera except for one individual who was immunocompromised because of solid organ transplantation. So, the negative result for IgM from the detection might have been affected of either the sensitivity of the test or the host’s immunological weakness due to the immunosuppressive drug [4, 74]. The efficiency of HEV RNA detection in feces (90%; 18 of 20 samples) was better than in serum (65%; 13 of 20 samples). This observation is similar to that involving HEV RNA detection in the other study [75].

### HEV genotyping using the ORF2 phylogeny

Using only ~ 900 bp of the capsid protein sequences might not involve enough phylogenetic signals for HEV systematics and evolution. This is because the mutations occurred randomly throughout the HEV genome [76, 77], and the tree reconstructed from the short sequences probably conflicted with the genome tree. The ORF2 phylogenies reconstructed from the ML and BI methods revealed the conflict topology against the genome phylogeny. This confirmed that the phylogenetic characters used in the dataset (568 bp after truncation of the unwanted regions) have less power for resolving HEV's systematic and evolution. However, the sequences are adequate for most of HEV genotype identification. HEV in each genotype were part of the monophyletic group except the genotype 3, which were not cluster the HEV isolated from rabbits within the clade of HEV genotype 3.

### HEV genotype 3

The genome phylogeny demonstrated that the monophyly of the HEV genotype 3 (Fig. 3). Within the genotype 3 clade, the HEV isolated from the non-human hosts (FJ906895, KJ013415, and JQ013791) were separated from the HEV genotype 3 that isolated from humans. The HEV isolated from human hosts was further split into two subclades, called the subgenotype 3.1 and 3.2 (Fig. 3). This branching pattern was also observed in the ORF2 phylogeny (Fig. 1). The Thai isolates of HEV appeared to be the genotype 3, especially the subgenotype 3.1 (16 of 18 isolates). However, the HEV genotype 3.2 was less frequently found among Thai population than the subgenotype 3.1 (2 of 18 isolates).

In comparisons using the current genotyping system [18], the subgenotype 3.1 gathered the genotypes 3e (AB248521), 3f (AB369687), and 3g (AF455784) within the clade. Contrary to the 3.1, HEV genotype 3.2 seems to have more genetic diversity. It contains the genotypes 3a (AF082843), 3b (AP003430), 3c (FJ705359), 3h (JQ013794), 3i (FJ998008), 3j (AY115488), 3k (AB369689), and 3l (JQ953664) in the tree [18, 78].

In conclusion, the HEV-genome based genotyping of the 18 Thai patients showed that they belonged to the genotype 3. Majority of those isolates belonged to the subgenotype 3.1, while a couple of isolates belonged to the subgenotypes 3.2. We also provided two HEV genomes from Thailand. The greater number of HEV genomes allows for explorations of mutations in the other ORFs. Improvement of the molecular phylogenetic systematics will enhance the accuracy of the HEV genotyping and evolution. Our knowledge from the tree can also apply to manipulating the HEV in public health and to providing the necessary information in molecular epidemiology for the prevention of HEV outbreaks.
